# Attributing extreme fire risk in Western Canada to human emissions

**DOI:** 10.1007/s10584-017-2030-0

**Published:** 2017-07-15

**Authors:** Megan C. Kirchmeier-Young, Francis W. Zwiers, Nathan P. Gillett, Alex J. Cannon

**Affiliations:** 1 Pacific Climate Impacts Consortium, University of Victoria, Victoria, BC V8W 2Y2 Canada; 2 Canadian Centre for Climate Modelling and Analysis, Environment and Climate Change Canada, University of Victoria, Victoria, BC V8W 2Y2 Canada; 3 Climate Research Division, Environment and Climate Change Canada, University of Victoria, Victoria, BC V8W 2Y2 Canada

**Keywords:** Event attribution, Fire weather, Extremes

## Abstract

Canada is expected to see an increase in fire risk under future climate projections. Large fires, such as that near Fort McMurray, Alberta in 2016, can be devastating to the communities affected. Understanding the role of human emissions in the occurrence of such extreme fire events can lend insight into how these events might change in the future. An event attribution framework is used to quantify the influence of anthropogenic forcings on extreme fire risk in the current climate of a western Canada region. Fourteen metrics from the Canadian Forest Fire Danger Rating System are used to define the extreme fire seasons. For the majority of these metrics and during the current decade, the combined effect of anthropogenic and natural forcing is estimated to have made extreme fire risk events in the region 1.5 to 6 times as likely compared to a climate that would have been with natural forcings alone.

**Electronic supplementary material:**

The online version of this article (doi:10.1007/s10584-017-2030-0) contains supplementary material, which is available to authorized users.

## Introduction

A wildfire near Fort McMurray, Alberta, Canada in early May 2016 burned almost 600,000 ha, but was most notable for displacing over 80,000 people (Government of Alberta 2016) and causing $3.5 billion in insured losses (IBC 2016). Following such an extreme event, questions arise regarding the extent to which human-induced climate change contributed to the event.

Wildfires in Canada burn approximately 2.1 million ha annually (Natural Resources Canada (NRCan) 2016). While large fires, classified as those burning more than 200 ha, constitute only 3% of all fires in Canada, they are responsible for about 97% of the area burned (Stocks et al. 2003). The occurrence and behavior of a fire is dependent on an ignition source, available fuels to burn, and weather conditions favorable for spread (Parisien et al. 2011), and can also be influenced by human suppression efforts. Williams and Abatzoglou (2016) review fire-modeling studies that assess climate influences on wildfire activity.

The Canadian Forest Fire Danger Rating System (CFFDRS; Stocks et al. 1989) is widely used to assess and predict wildfire risk and behavior across northern North America and is also applied in many other regions around the globe. CFFDRS is composed of the Canadian Forest Fire Weather Index (FWI) System (Van Wagner 1987), which uses weather conditions to calculate fire potential, and the Fire Behavior Prediction (FBP) System (Forestry Canada Fire Danger Group 1992), which uses information from the FWI and evaluates the behavior of an ignited fire for different fuel types.

As wildfires have numerous impacts, many studies have investigated changes to fire risk under future climate projections (Flannigan et al. 2009). Through lengthening of the fire season (Flannigan et al. 2013; Liu et al. 2013), increased days with spread potential (Wang et al. 2015), increased fire risk (de Groot et al. 2013), or projected increases in the number of fires (Wotton et al. 2010; Krawchuk et al. 2009) and area burned (Balshi et al. 2009; Flannigan et al. 2005), a heightened fire risk is expected in Canada and the USA under scenarios with increasing anthropogenic greenhouse gases. Jolly et al. (2015) used reanalyses to demonstrate an increase in fire season length has already been seen globally through 2013. Flannigan et al. (2016) estimated up to a 15% increase in precipitation is needed to offset each degree of warming in terms of the FWI indices and thus increasing temperatures will result in more days with high fire potential. Increased fire risk has the potential to exceed the capabilities of current fire management agencies (Podur and Wotton 2011; Flannigan et al. 2009).

To assess the anthropogenic influence on fire risk in western Canada, we utilize an event attribution framework (NASEM 2016), which aims to quantify the influence of anthropogenic forcings on the frequency (our focus in this paper) or magnitude of specific classes of extreme events. The methodology generally involves comparing the probability of a particular event’s occurrence in a world with observed emissions (ALL forcing = natural (NAT) + anthropogenic (ANT)) and in a counterfactual world (NAT only). Because direct observations of the counterfactual world are unavailable, event attribution studies typically rely on large ensembles of climate model simulations. Only a few studies have pursued the attribution of fires and wildfire risk directly, though attribution of increased temperatures and drought events can also lend insight into fire risk. Using the strong relationship between temperature and area burned, Gillett et al. (2004) detected an anthropogenic contribution to increasing area-burned trends in Canada. With a large ensemble from CESM1, Yoon et al. (2015) found a deviation in drought and fire risk between simulations that included anthropogenic forcings and those with only natural variability beginning in the 1990s and thus attributed an increased fire risk in California to human emissions. Abatzoglou and Williams (2016) found that anthropogenic signals account for approximately half of the increasing trends in fuel aridity and fire season length in the western USA. Finally, Partain et al. (2016) demonstrated that the fuel conditions leading to the severe 2015 fire season in Alaska were more likely with ALL forcings than in a counterfactual world.

The goal of this paper is to use an event attribution perspective to quantify the influence of anthropogenic forcings on extreme wildfire risk in a region of western Canada that includes Fort McMurray. Fourteen metrics are utilized to define extreme fire risk on a fire-season basis for the 2011–2020 climate and the probabilities of these extreme fire seasons are compared between a scenario with ALL forcings and a counter-factual scenario with only NAT forcings. In Section 2, we introduce the observations, model, and region used in this analysis. Section 3 provides an overview of CFFDRS and the calculation of its indices. The event attribution methodology, event definitions, and results are presented in Section 4, with conclusions in Section 5.

## Data

### Observations and region

The Global Fire Weather Database (GFWED; Field et al. 2015) is a gridded dataset of daily FWI indices. The data are available on the 1/2° by 2/3° grid of the MERRA reanalysis (Rienecker et al. 2011), beginning in 1980. GFWED provides the four main FWI System indices as well as the daily input weather data for the FWI-system standard of local noon values. The temperature, relative humidity (RH), and wind speed inputs for the FWI indices calculated here are from GFWED. Precipitation is obtained from the Multi-Source Weighted-Ensemble Precipitation (MSWEP) dataset (Beck et al. 2016), which combines surface-based and remotely-sensed observations and reanalysis products to create a global 3-hourly precipitation dataset on a 0.25° grid. The data were aggregated to 24-h accumulations as close to the GFWED data as possible and interpolated to the MERRA grid. See the supplementary material for more discussion of the choice of precipitation dataset. The observations are mainly used for bias-correcting the model data.

**Fig. 1:**
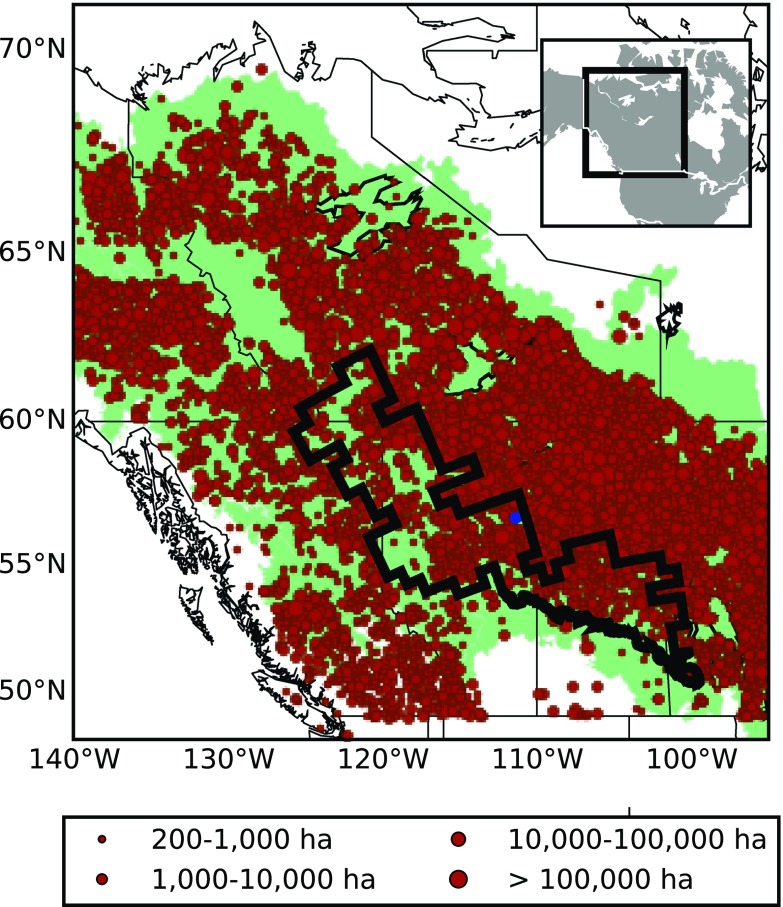
Western Canada region used in this study. The *green* shading represents the Boreal zone (Brandt 2009). *Red* points are ignition locations of large fires (> 200 ha) for the 1980–2014 period from the CNFDB, scaled by fire size. *Black* region is HFR9 and the *blue* dot is Fort McMurray

We use the homogeneous fire regime (HFR) zones defined by Boulanger et al. (2012), who used a cluster analysis of fire characteristics and climatologies to refine the eco-classifications of the Ecological Stratification Working Group (ESWG) (1996) to regions more suited to wildfire analyses. The HFR zones in western Canada were numbered by the authors and the region containing Fort McMurray was selected (Fig. 1). This region, covering approximately 5.7x 10^7^ ha or 267 GFWED grid boxes, will be referred to as HFR9 herein.

### Model

The model simulations are from large ensembles of the Canadian Earth System Model version 2 (CanESM2; Arora et al. 2011; Fyfe et al. 2017). The first large ensemble contains 50 ensemble members (also referred to as realizations) with ALL forcing and the second contains 50 ensemble members with NAT forcing. NAT forcing includes solar and volcanic influences, while ALL forcing is a combination of natural forcing and the anthropogenic components (greenhouse gases, aerosols, land use, etc). The NAT simulations are available through 2020 and ALL simulations through 2100, with years beyond 2005 forced with RCP8.5. Herein, ALL data are not used beyond 2020 and there is a negligible difference between RCPs for this period (van Vuuren et al. 2011). Daily values of maximum temperature, mean wind speed, and total precipitation were acquired from the model output; RH was calculated using the model-supplied specific humidity. More details on the CanESM2 ensemble can be found in the supplementary material.

The topography of the region, namely the Western Cordillera, can have an impact on the local weather and thus on the fire indices and these effects may not be captured adequately with the coarse resolution (2.81°) of CanESM2. Other studies have employed simple downscaling models (Flannigan et al. 2013; Wang et al. 2015) to increase resolution or statistical models to relate model outputs to the number or area of fires (Wotton et al. 2010; Balshi et al. 2009). Here, the large ensemble output was downscaled to the resolution of the GFWED data and then bias corrected following the methodology of Cannon (2017), which bias corrects the marginal distributions and maintains the multivariate dependence structure between the four variables (tair, RH, wspd, prcp). If debiased separately, the relationship between weather variables could be altered, which would have implications for the calculation of the CFFDRS indices (Cannon 2017). The downscaling/bias correction considers internal variability between realizations and maintains the separation between the ALL and NAT responses; the procedure is described in more detail in the supplementary material.

## CFFDRS

### Indices

The FWI System (Van Wagner 1987; see Table 1) uses weather variables to assess the risk of fire ignition and spread. The system includes three indices (FFMC, DMC, and DC) that describe the moisture available in fuels of increasing depths and the FWI index, which provides a summary measure of the fire potential. For all indices, larger values indicate higher fire risk. The FWI system indices are calculated daily using observations at local noon and depend on the previous day’s index. We use the recommended values (NRCan 2016) to initiate the calculations on the first day of the fire season and ignore overwintering adjustments. Indices from the observations and downscaled model simulations were calculated by grid box using the same routine. More detail regarding the interpretation of the indices and their use by fire managers is available in Wotton (2009).

**Table 1:** List of fire weather and behavior indices from the CFFDRS and the input variables they depend on, including air temperature (tair), relative humidity (relh), and wind speed (wspd) at local noon and 24-h precipitation (prcp)

System	Index		Description	Extreme	Input variables
FWI	Fine Fuels Moisture Code	FFMC	moisture in surface fuels; memory: 3 days; prcp threshold: 0.5 mm	91	tair ^+^, relh ^−^, prcp ^−^ wspd ^+^
FWI	Duff Moisture Code	DMC	moisture in decaying litter, upper layers; memory: 14 days; prcp threshold: 1.5 mm	60	tair ^+^, relh ^−^, prcp ^−^
FWI	Drought Code	DC	moisture in deep layers, large debris; memory: 51 days; prcp threshold: 2.8 mm	425	tair ^+^, prcp ^−^
FWI	Initial Spread Index	ISI	potential fire spread rate	15	FFMC, wspd ^+^
FWI	Buildup Index	BUI	potential fuel available	90	DMC, DC
FWI	Fire Weather Index	FWI	summary of fire potential	30	BUI, ISI
FWI	Daily Severity Rating	DSR	rescaled FWI for categorical interpretation	15	FWI
FBP	Surface Fuel Consumption	SFC	amount of fuel consumed by the fire (kg m ^−2^)	4	FFMC, BUI, fuel type
FBP	Rate of Spread	ROS	rate (m min ^−1^) at which the fire head (leading edge) moves	18	ISI, BUI, fuel type
FBP	Head Fire Intensity	HFI	intensity (kW m ^−1^) at the fire head	10,000	ROS, SFC

Superscripts indicate whether increasing values of the input weather variables result in increased (+ ) or decreased (−) values of the index. Descriptions from (Wotton 2009), memory from (Field et al. 2015), extreme values from (NRCan 2016)

The FBP System incorporates some of the FWI indices to provide more detail on the expected behavior of an ignited fire, with the calculations (Forestry Canada Fire Danger Group 1992) dependent on specific fuel types (Nadeau et al. 2005). The three main fuel types for HFR9 are Boreal spruce (C2), Lodgepole pine (C3), and leafless aspen (D1) (S. Taylor, personal communication). We focus on C2 due to its prevalence in northern Alberta (Nadeau et al. 2005), but include results for C3 and D1 in the supplementary material.

**Fig. 2:**
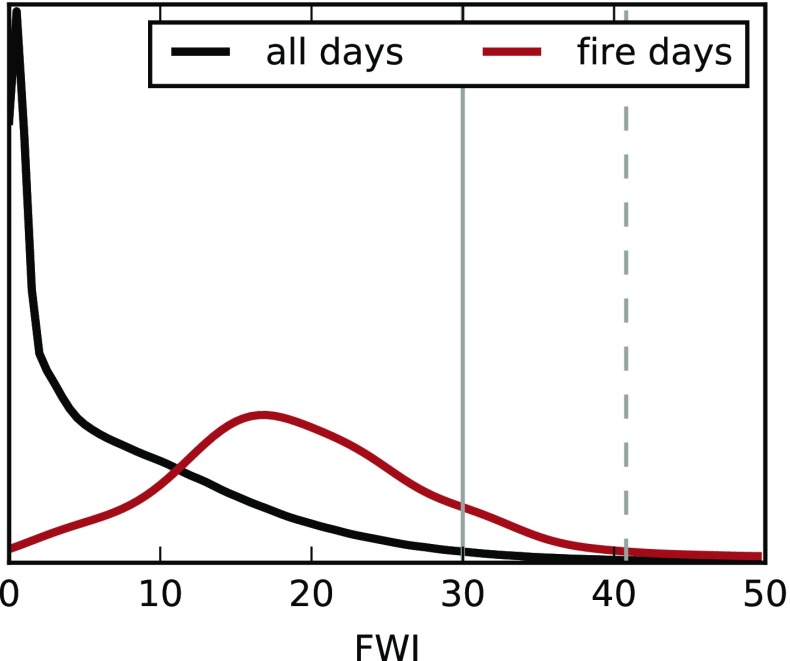
Density curves for all Fire Weather Index (FWI) values during the fire season (MJJAS) by grid box in HFR9 using the GFWED-MSWEP data for 1980–2014 (*black*) and for only grid boxes where a large fire ignited, using the maximum FWI value in the first four days (*red*). *Vertical bars* indicate extreme values of the index defined by NRCan (*solid*) and the value at the time of ignition of the Fort McMurray fire (*dashed*). About 800 values went into the *red curve* and on the order of 10^6^ values are summarized by the *black curve*

An example of the relationship between the FWI and fires in HFR9 is shown in Fig. 2, which compares density curves of the FWI values for all days and all gridboxes with FWI values for days and gridboxes corresponding to large fires. Fire data were acquired from the Canadian National Fire Database (CNFDB; Stocks et al. 2003). The maximum FWI in the first four days following ignition is assigned to each fire; this window was chosen based on when a fire consumes most of its fuel (Amiro et al. 2004). In general, large fires occur on days with greater fire potential (larger FWI). There are many days every year that experienced extreme fire risk but either lacked an ignition source or any ignited fires were suppressed quickly. Additional analyses of the relationship between large fires and the FWI and FBP indices can be found in Amiro et al. (2004); Kiil et al. (1977).

### Fire season

There is no standard definition for the fire season. The official NRCan recommendation (Turner and Lawson 1978) is to begin the calculation of the FWI System indices after three consecutive days without snow cover in regions that experience significant winter snow cover, or after three consecutive days with noon temperatures over 12 °C. All of the grid boxes in HFR9 see significant winter snow cover.

For observations, following the GFWED (Field et al. 2015), a grid box is snow covered if snow depth is greater than 1 cm. The fire season start dates were calculated after three consecutive days without snow cover, beginning 01 March. The grid boxes in HFR9 generally start their fire seasons in late April or early May (Fig. S1a), whereas regions farther north or at higher elevations start later in the year. The fire season ends with the first snowfall, defined as the first day after 01 July with snow cover, which occurs in September for much of the region (Fig S1b). The resulting fire season length averages approximately 150 days (Fig. S1c) in HFR9.

For CanESM2 simulations, a simple statistical model was derived to predict the fire season start date for each year based on mid-spring growing degree days; more information regarding its calibration can be found in the supplementary material. The fire season end date was defined as the first day after 01 July where a grid box reported precipitation over 0 mm and a noon temperature below 5 °C.

## Event attribution

### Setting up the event attribution

A detection and attribution analysis (Bindoff et al. 2013) was used to assess whether anthropogenic influence has had a discernible effect on the base climate state in the larger western Canada region. Evidence that anthropogenic influence has altered the base state would increase confidence in the attribution of extreme events, which can be viewed as departures from this altered base state (NASEM 2016). Mean temperature was chosen due to its robust detection globally (Bindoff et al. 2013) and throughout many regions (Stott 2003; Zhang et al. 2006), its observational coverage, and its well understood relationship to increased greenhouse gases. As there can be smaller signal-to-noise ratios at the regional level and among other variables, it can be more difficult to provide a robust attribution (Stott et al. 2010). Performing a detection and attribution analysis for temperature over a larger region and longer time period helps to reduce the impact of noise on such analyses.

Observed monthly temperatures over land areas from the CRU-TS3 dataset (Harris et al. 2014) on a 0.5° grid were averaged over western Canada (Fig. 1). Similarly, monthly average temperatures from CanESM2 ALL and NAT realizations were averaged over the land grid boxes for this region. A detection and attribution analysis was performed for the longest period available (1960–2014); a thorough description of the methods applied here can be found in Kirchmeier-Young et al. (2016).

The ALL forcing signal was detected in the observations (Fig. S2) for the fire season (MJJAS), though CanESM2 overestimates the warming during this period. After scaling the model response to be consistent with the observations, the result is an attributed warming trend of about 1 °C over the period for which the FWI indices can be calculated (1980-2014). As anthropogenic forcing has had a demonstrable influence on the region, it is reasonable to pursue an event attribution analysis for a more localized region and for other variables that, while influenced by temperature change, likely present smaller signal-to-noise ratios.

### Event definitions

A key first step for event attribution is framing the attribution question (NASEM 2016), which includes determining the spatial and temporal characteristics and climate variable to define the event of interest. Although the events chosen for attribution analyses are typically inspired by societally-relevant extreme events, selection bias becomes a concern when using an event definition that is too specific (e.g., observed extreme at a point location). Furthermore, using multiple event definitions can increase the robustness of event attribution results (NASEM 2016).

We use the class type of event definition (NASEM 2016) by defining an event as all possible outcomes for which a particular metric exceeds a chosen threshold (Table 2). First, we define a class of events for each FWI index by requiring the 90th percentile of daily index values for each fire season to exceed an NRCan (2016) defined “extreme” threshold. FWI index percentiles have been used in other studies (Wotton et al. 2010; Parisien et al. 2011; Wang et al. 2015) and are a better indicator of extreme fire days than a measure of central tendency. The NRCan thresholds are defined for all of Canada and may not completely characterize local extremes. For reference, the maximum value of each index during the first 4 days of the Fort McMurray fire, based on data from the Fort McMurray airport weather station, was FFMC-95, DMC-56, DC-370, ISI-21, BUI-81, FWI-40 (M. Flannigan, personal communication). Bearing in mind the inherent difference between station and gridded observations, these values correspond, respectively, to the > 99, 94, 89, > 99, 95, > 99th percentiles in the corresponding grid box of GFWED data.

**Table 2:** Event attribution results for many extreme fire risk metrics

Event	*p*_0_	*p*_1_	PN	PS	RR
Fire Season 90th percentile					
FWI > 30	< 0.01	0.03	0.83	0.03	5.97
FFMC > 91	0.05	0.15	0.66	0.11	2.95
DMC > 60	0.23	0.36	0.35	0.17	1.55
DC > 425	0.39	0.56	0.31	0.29	1.45
ISI > 15	< 0.01	< 0.01	-	-	-
BUI > 90	0.15	0.26	0.44	0.14	1.78
Significant spread potential					
> 38 days	0.03	0.12	0.74	0.09	3.90
> 25%	0.04	0.11	0.65	0.08	2.82
ROS p90 > 18 [C2]	< 0.01	< 0.01	1.00	< 0.01	10^9^
Fire Intensity Classes					
> 38 days Class 5/6 [C2]	0.16	0.37	0.55	0.24	2.22
> 76 days Class 5/6 [C2]	< 0.01	< 0.01	0.96	< 0.01	22.52
HFI p90 > 10,000 [C2]	0.08	0.23	0.63	0.16	2.72
Fire Season					
Fire season starts by 15 Apr	0.09	0.19	0.52	0.11	2.10
Fire season ends after 31 Sep	0.09	0.25	0.65	0.18	2.86
Fire season > 165 days	0.05	0.20	0.76	0.16	4.12

Values are rounded to two decimal places for display purposes. See Table S1 for uncertainties. Attribution metrics are not calculated for ISI as the extreme threshold exceeds any regional values realized in either the ALL or NAT simulations

We also considered events defined in terms of days with significant spread potential, by using the 90th percentile value of the ROS (Rate of Spread) and also the definition of Wang et al. (2014) that determines spread days in a rain-free period as those with FWI (Fire Weather Index) ≥19 and DMC (Drought Moisture Code) ≥20. Spread days are expressed as the number of days per season or the percentage of the fire season length, with thresholds for an extreme season being 38 days (25% of the climatological mean season length) or 25%, respectively. We also use the fire season 90th percentile of Head Fire Intensity (HFI) and metrics characterized by the number of days in fire intensity classes 5 and 6 (HFI > 4,000; NRCan 2016). Finally, we look at metrics describing the fire season, including start and end dates and the length of the season.

### Methodology and metrics

**Fig. 3:**
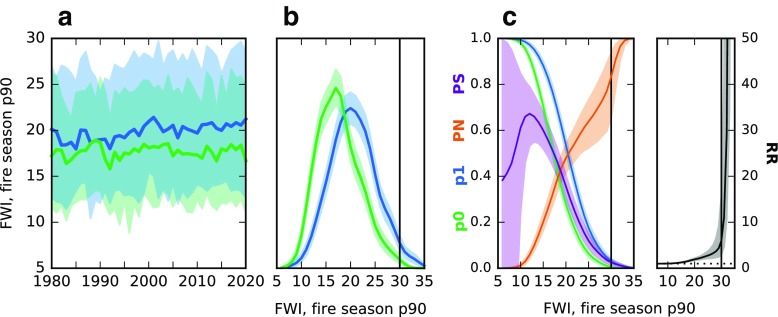
**a** Time series of fire season 90th percentile values of the Fire Weather Index (FWI) for the ALL forcing ensemble mean in *blue* and NAT forcing ensemble mean in *green*. Shading represents the 5th–95th percentile range across the ensemble. **b** Density plots for 2011–2020 for ALL in *blue* and NAT in *green*, pooling values from the ensemble members and using a Gaussian kernel density estimator. A non-parametric 90% uncertainty range is *shaded*, determined through bootstrapping. The *vertical bar* represents the threshold for an extreme value; for comparison, the Fort McMurray station saw an FWI value of 40 on the day of fire ignition. **c** Plots of p0, p1, PN, PS, and RR for a fire season 90th percentile value more extreme than the threshold on the horizontal axis. The probabilities (p0 and p1) are determined by empirically integrating the density curves and the shaded uncertainty ranges are a result of the uncertainty on the density curves

For each of the metrics discussed above, the probabilities of an event occurring under ALL and under NAT forcing were calculated by pooling the values from all realizations for a chosen decade. Each metric was calculated by grid box and then averaged across HFR9. An example using the 90th percentile of the FWI is shown in Fig. 3. For each realization, each year and each grid box, the 90th percentile of daily FWI values is determined and averaged over HFR9; the result is a value for every year and every realization. Time series of the 90th percentile of FWI (Fig. 3a) show that as time progresses, the separation of the ALL (blue) and NAT (green) ensemble means increases, with a slight increasing trend under ALL forcing and no trend under NAT forcing.

Choosing the current decade, 2011–2020, the values from each year and each realization are pooled together (500 years total) and density curves estimated (Fig. 3b). The density curve for ALL (blue) is shifted toward slightly larger values of FWI than the NAT curve. These densities are then used to calculate the probability of a particular event; *p*_0_ is the probability under NAT forcing and *p*_1_ the probability of the same event under ALL forcing (Fig. 3c). Numerous thresholds to define events (horizontal axis) are used. Both *p*_0_ and *p*_1_ decrease with increasing severity of FWI values, but *p*_1_ decreases more slowly as the extreme events are more likely with ALL forcing.

The probabilities are used to calculate three event attribution metrics:

1 $$ PN = FAR = 1 - \frac{p_{0}}{p_{1}} $$

2 $$ PS = 1 - \frac{1 - p_{1}}{1 - p_{0}} $$

3 $$ RR = \frac{p_{1}}{p_{0}} $$

The probability of necessary causality (PN) and the probability of sufficient causality (PS) were introduced in Hannart et al. (2016). PN describes the probability that ALL forcing is a necessary cause of the particular event; that is, that ALL forcing is required for the event’s occurrence. PS describes the probability that ALL forcing is sufficient for the event, such that a scenario with ALL forcing will see the occurrence of this event every time. Any negative values of PN or PS are set to 0. PN is also the fraction of attributable risk (FAR; Stott et al. 2004), which describes the fraction of the risk of an event’s occurrence contributed by the anthropogenic (ANT) component. Finally, the risk ratio (RR) describes how many times as likely the event occurrence is with ALL than with NAT.

The resulting curves for the event attribution metrics are shown in Fig. 3c. PN increases with increasing severity of FWI values. A PN value of approximately 0.8 for a fire season 90th percentile value of the FWI exceeding 30 means that 80% of the risk of this event is due to anthropogenic (ANT) forcing, there is an 80% chance that ANT forcing is required for this event to occur, or eight out of ten occurrences of this event would not have happened with only NAT forcing. The PS values are small for the more extreme FWI thresholds, as such events are rare with both forcing scenarios (see Fig. 3b). Finally, the RR is greater than 1 (the event is more likely under ALL forcing) for all FWI thresholds. RR values increase rapidly for the more extreme values of FWI. An RR of 10 would imply the occurrence of that event is 10 times as likely under ALL forcing than under NAT forcing.

### Results

**Fig. 4:**
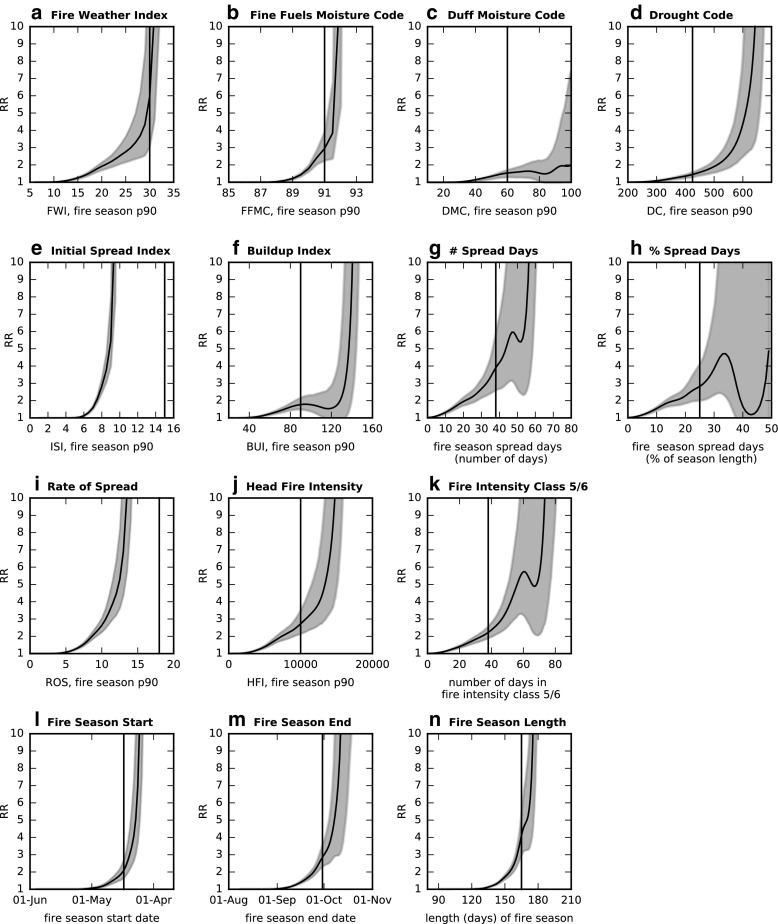
The risk ratio (RR) for many metrics based on a 2011–2020 climate. Values are for an event more extreme than that indicated on the *horizontal axis* and the *vertical bar* represents the threshold for an extreme value (see Table 2 and Table S1). The uncertainty range for each RR curve is *shaded* and was calculated using a bootstrapping method. The FBP metrics in panels **(i)–(k)** use the C2 fuel class

All of the metrics show density curves for ALL forcing that favor more extreme values compared to NAT forcing for 2011–2020 (Fig. S3). For the FWI indices, this is likely due to the strong signal seen in temperature and to a lesser extent the difference in wind speed between the two forcing scenarios (Fig. S4, S5). The extreme thresholds (vertical bars) are rare events for many of the indices, resulting in small values of *p*_1_ and even smaller values of *p*_0_ for these events (Table 2). For the FWI indices, the RR values range from about 1.5 to 6 times as likely under ALL forcing and the confidence intervals on these values are generally small (Fig. 4).

The significant spread days show similar results between the counts and percentage metrics, with approximately 70% of the event risk due to anthropogenic forcings (Table 2). Under ALL forcing, it is almost three times as likely for a fire season to have significant spread potential on more than a quarter of its days. Using the 90th percentile value of the ROS (Rate of Spread) sees smaller probabilities for events exceeding the extreme threshold and much stronger attribution results, with a PN value indicating ANT forcing is a necessary cause.

Events for the 90th percentile of the HFI (Head Fire Intensity) and the number of days in the top fire intensity classes are several times as likely under ALL forcing (Table 2). These results are sensitive to fuel type (Fig. S7) though it is expected that a spruce forest (C2) will have more burn potential than one with leafless aspen (D1).

An increased fire season length under ALL forcing (Fig. S3m) is consistent with other studies that demonstrated extended fire seasons under future scenarios including increased anthropogenic emissions (Flannigan et al. 2013; Liu et al. 2013). There is a 76% chance that ANT forcing is necessary for a fire season exceeding 165 days and such an event is 4 times as likely than with NAT forcing alone (Table 2). This is influenced by both a later end date and earlier start date to the fire season.

Generalizing to other thresholds, RR (Fig. 4) and PN (Fig. S8) curves are shown for an event more extreme than the given index value. Consistent with the densities (Fig. S3), all metrics see increasing PN values for more extreme thresholds, indicating an increased contribution of ANT forcing to the occurrence of such events. This is consistent with increasing RR values for more extreme thresholds. PN reaches 1.0 in the upper tail of the ALL distribution for most metrics with very large RR values, which would implicate ANT forcing as a necessary cause. Although the exact RR values can be sensitive to the estimation of very small probabilities, such events would be considerably more likely to occur with ALL forcing than with NAT forcing.

## Discussion and conclusions

This study uses a single model ensemble; although the large number of realizations should adequately represent internal variability, detection and attribution analyses can benefit from multi-model ensembles (Hegerl and Zwiers 2011) that reduce the influence of a particular model’s biases. The simulations used here were debiased relative to a reanalysis product, which requires assumptions about its representativeness for the region. The downscaling and bias correction routines may also introduce their own sources of error. Additionally, the bias correction does not fully correct the trends and the model used here may overemphasize the warming trend for this region, which would result in over-confident attribution. Limited coverage of observations in this region presents challenges for evaluating reanalysis or model performance.

Using several event definitions strengthens an event attribution result (NASEM 2016) and those chosen here include numerous ways to represent extreme fire risk and potential. These event definitions were chosen with the consultation of a fire scientist (S. Taylor, personal communication) and represent extreme fire risk from a climate perspective; at a local level, fire managers may require different metrics and thresholds to best define fire risk (Wotton 2009). Furthermore, this analysis does not consider changes to forest health or composition as a result of climate change (Gauthier et al. 2015). Changing forest and fire management practices can also impact future fire activity.

Despite these caveats, it was shown that ALL forcing produces an increase in the risk of extreme fire potential compared to NAT forcing alone, using many different metrics. The ALL forcing responses saw longer fire seasons, with more days with significant spread potential and/or conditions suitable for high-intensity fires, and also greater values of the FWI indices designed to represent fuel availability and fire potential. For the majority of these metrics and during the current decade, ALL forcing is estimated to have made extreme fire risk events in the HFR9 region 1.5 to 6 times as likely than would have been the case under only NAT forcings. Thus, the Fort McMurray fire of May 2016 occurred in a world where earlier and longer fire seasons are more likely; where there is an increased risk of extreme fire potential (based on the FWI indices); and a larger number of potential spread days that can result in the growth of a large fire. Many metrics of fire potential showed elevated risk as a result of the combination of natural variability and human emissions.

## Electronic supplementary material

Below is the link to the electronic supplementary material.

(PDF 1.11 MB)
