# Supplementary material for: Attributing extreme fire risk in Western Canada to human emissions
Source: Clim Change. 2017 Jul 15;144(2):365–79. doi: 10.1007/s10584-017-2030-0 (PMC6961511; doi:10.1007/s10584-017-2030-0)
Supplement: Supplementary file 1 — (PDF 1.11 MB) [file 10584_2017_2030_MOESM1_ESM.pdf]

Supplementary Material

Attributing Extreme Fire Risk in Western Canada to Human Emissions

M. C. Kirchmeier-Young, F. W. Zwiers, N. P. Gillett, A. J. Cannon

1 Precipitation observations

The Global Fire Weather Database (GFWED; Field et al 2015) offers three different precipitation datasets, one from reanalysis and two based on the interpolation of gauge observations. The station density in western Canada is low (e.g., see Figure 10 in Field et al 2015), which presents challenges for the calculation of gridded gauge-based observations datasets in this region. The interpolation of sparsely located gauges leads to higher uncertainties in un-gauged locations, particularly in regions of complex terrain. Several precipitation datasets were investigated over this western Canada region and MSWEP (Multi-Source Weighted-Ensemble Precipitation; Beck et al 2016) was chosen for its representation of the topographical features with both the general climatology and extreme events. Although this dataset can suffer from too many days with very light precipitation (not shown), an issue common to reanalyses and models (Sun et al, 2006), the calculation of the FWI System indices does not consider rainfall below 0.5 mm.

2 Model

The Canadian Earth System Model version 2 (CanESM2; Arora et al 2011) was used to produce a large ensemble as part of the Canadian Sea Ice and Snow Evolution (CanSISE) Network project. The CanESM2 submission to CMIP5 contained 5 ensemble members with simulations beginning in 1850; the different realizations were branched off from the pre-industrial control run every 50 years. For the large ensembles (Fyfe et al, 2017), each of the 5 historical ALL and NAT forcing ensemble members was branched into 9 additional members in 1950, resulting in 50 member ensembles each under ALL forcing and NAT forcing. In 1950, the seed of a random number generator included in the parameterization of a cloud process was changed to produce the different realizations.

3 Downscaling procedure

The topography of the region, namely the Western Cordillera, can have an impact on the local weather and thus on the fire indices and these effects may not be captured adequately with the resolution of CanESM2. Here, the large ensemble output was downscaled to the resolution of the GFWED data. This was done for each variable and for each realization by first calculating anomalies on the original, large-scale grid relative to an ensemble mean monthly climatology. These anomalies were bi-linearly interpolated to the target grid and the climatology of the observations added by month. For precipitation, a ratio with climatology was used instead of a difference due to the presence of many rain-free days. Hard bounds were enforced for RH [0,100] and wind speed $[0,\infty)$.

The data were then debiased following the methodology of Cannon (2017), which employs a multivariate quantile mapping bias correction procedure that maintains the multivariate dependence structure between the four variables (tair, RH, wspd, prcp), as well as the forced response of the climate model (e.g., absolute changes in quantiles for tair and RH and relative changes in quantiles for wspd and prcp relative to the ALL baseline). If debiased separately, the relationship between weather variables could be altered, which would have implications for the calculation of the CFFDRS indices. This is indicated in split-sample verification results for FWI system indices (Cannon, 2017) based on evaluation runs of CanRCM4 (Scinocca et al, 2016), a regional climate model that uses the same physical parameterizations as CanESM2.

The multivariate quantile mapping algorithm is based on the N-dimensional probability distribution transform (N-pdft) proposed by Pitié (2005, 2007). The multivariate distribution of the climate model simulations is transformed to match the distribution of historical observations by iteratively applying a

random orthogonal rotation to the datasets followed by univariate quantile mapping of the rotated marginal distributions. The additional rotation step provides linear combinations of the original variables – rather than each original variable separately – to the univariate quantile mapping bias correction. When these two steps are combined in sequence and repeated, correction of the multivariate distribution becomes possible. The algorithm is proven to converge with random rotation matrices and a multivariate Gaussian target (Pitié et al, 2007), which means that any distribution can be mapped to another by using the multivariate Gaussian as a pivot. Empirical evidence suggests, however, that the intermediate Gaussian is unnecessary and that direct mapping between arbitrary continuous distributions is possible.

In Cannon (2017), the N-pdft algorithm is extended for use with climate model simulations by further ensuring that the post-processed outputs preserve the climate change signal of the underlying climate model in the projection period, subject to multivariate bias correction of the empirical copula in the historical calibration period. This is done by first applying a change-preserving form of univariate quantile mapping (Cannon et al, 2015) to each variable of the climate model projection dataset and saving the outputs. Next, the multivariate N-pdft algorithm is used to bias correct both the historical calibration and climate model projection datasets. Finally, the change-preserving quantile mapping outputs in the projection period are reordered, following Cannon (2016), according to the ordinal ranks of the corresponding variable from the N-pdft algorithm. This maintains the preservation of trends from the quantile mapping algorithm and the rank dependence structure from the N-pdft algorithm. As described below, the change-preservation property allows the NAT realizations to maintain their separation from the ALL response. More details and an implementation of the algorithm are provided at https://cran.r-project.org/package=MBC

The bias correction procedure is trained with a baseline period of 1980-2014 to include all available observations and then applied over 1980-2020; each ALL and NAT realization is debiased using a different ALL realization for calibration to account for internal variability. Failure to account for internal variability when applying bias correction algorithms will artificially reduce ensemble spread (Kim et al, 2016). The combined downscaling/bias correction approach maintains the separation between the ALL and NAT responses; before downscaling, the NAT anomalies are calculated relative to the ALL ensemble mean climatology. For each of the ALL and NAT realizations, the multivariate quantile mapping then corrects marginal distributions and multivariate dependence structure using an ALL calibration realization as the model reference in the N-pdft algorithm. Subject to the N-pdft corrections needed to transform the ALL calibration distribution to the observed distribution, simulated differences between empirical quantiles for the NAT or ALL realization of interest and the ALL calibration realization are also preserved following Cannon (2016). Furthermore, the bias correction routine also corrects for any difference between the maximum temperature and daily averaged values of the other variables and the local noon values requested for the FWI System calculations and provided by GFWED.

4 Fire season statistical models

Instead of downscaling and bias correcting snow information from the coarse-resolution model realizations, a simple statistical model was derived to predict the fire season start date for each year. The multiple linear regression model was calibrated with the observations, using the snow-based fire season start date (day of year) as the predictand. A cross-validation procedure was used to select the predictors: a year was removed from the time series, the model was fit for all predictor combinations with the remaining years and then applied to the removed year, and this procedure was repeated for all years. The Akaike information criterion (AIC) was used to determine the best predictor set. Although the model is fit and applied by grid box, the predictors were chosen common to all grid boxes in HFR9. Tested predictors included spring mean temperature, total winter precipitation, snow cover, growing degree days, and the fire season start date using a temperature-based approach. The final model includes only the growing degree days over a 0°C threshold (calculated with noon temperature) at the end of April.

There are fewer predictor options to predict the date of the return of snow cover and regression models tested performed very poorly. Hence, the fire season end date was defined as the first day after 01 July where a grid box reported precipitation over 0 mm with a noon temperature below 5°C. Several thresholds were tested for both temperature and precipitation and the pair that resulted in the lowest root mean square error for the observations compared to the snow-based date was chosen.

5 Wind speed sensitivity analysis

A sensitivity analysis was performed to compare the influence of increases in wind speed with increases in temperature on the FWI and FBP indices. Figure S6 shows the density curves of fire season 90th percentile values for NAT forcing, as well as similar curves calculated with adjusted temperatures and also with adjusted wind speeds. For the adjusted inputs, all variables were taken from the NAT simulations, but the temperatures (and separately, the wind speeds) were shifted by the difference in mean values between the ALL and NAT simulations. Essentially, the FWI and FBP indices were recalculated using tair values of magnitudes seen in the ALL scenario, but maintaining the sequence of events and keeping all other input variables at their NAT values. This was repeated changing wind speed to ALL magnitudes and holding tair, relh, and prcp at NAT values. Only indices that depend on wind speed were included. The ISI is most affected by changes in wind speed and shows similar curves for temperature and for wind speed changes, both of which are shifted towards more extreme values than the NAT simulations. For all other indices, the temperature change has a stronger influence on the FWI and FBP indices than the wind speed change.

The difference in wind speed between the ALL and NAT responses appears to be due to land-use change early in the 20th century. Figure S6 shows a comparison of fire season mean wind speeds from six different forcing scenarios.

References

Arora VK, Scinocca JF, Boer GJ, Christian JR, Denman KL, Flato GM, Kharin VV, Lee WG, Merryfield WJ (2011) Carbon emission limits required to satisfy future representative concentration pathways of greenhouse gases. Geophys Res Lett DOI 10.1029/2010GL046270

Beck HE, van Dijk AIJM, Levizzani V, Schellekens J, Miralles DG, Martens B, de Roo A (2016) MSWEP: 3-hourly 0.25°global gridded precipitation (1979-2015) by merging gauge, satellite, and reanalysis data. Hydrol Earth Syst Sci Disc 21:589–615, DOI 10.5194/hess-21-589-2017

Cannon AJ (2016) Multivariate Bias Correction of Climate Model Outputs: Matching Marginal Distributions and Inter-variable Dependence Structure. Journal of Climate 29:7045–7064, DOI 10.1175/JCLI-D-15-0679.1

Cannon AJ (2017) Multivariate quantile mapping bias correction: An N-dimensional probability density function transform for climate model simulations of multiple variables. Climate Dynamics DOI 10.1007/s00382-017-2580-6

Cannon AJ, Sobie SR, Murdock TQ (2015) Bias correction of GCM precipitation by quantile mapping: How well do methods preserve changes in quantiles and extremes? Journal of Climate 28(17):6938–6959, DOI 10.1175/JCLI-D-14-00754.1

Field RD, Spessa AC, Aziz NA, Camia A, Cantin A, Carr R, de Groot WJ, Dowdy AJ, Flannigan MD, Manomaiphiboon K, Pappenberger F, Tanpipat V, Wang X (2015) Development of a Global Fire Weather Database. Natural Hazards and Earth System Science 15:1407–1423, DOI 10.5194/nhess-15-1407-2015

Fyfe JC, Derksen C, Mudryk L, Flato GM, Santer BD, Swart NC, Molotch NP, Zhang X, Wan H, Arora VK, Scinocca J, Jiao Y (2017) Large near-term projected snowpack loss over the western United States. Nature Communications 8:14,996, DOI 10.1038/ncomms14996

Kim KB, Kwon HH, Han D (2016) Precipitation ensembles conforming to natural variations derived from a regional climate model using a new bias correction scheme. Hydrology and Earth System Sciences 20:2019–2034, DOI 10.5194/hess-20-2019-2016

Pitié F, Kokaram AC, Dahyot R (2005) N-dimensional probability density function transfer and its application to colour transfer. Proceedings of the IEEE International Conference on Computer Vision II(November 2005):1434–1439, DOI 10.1109/ICCV.2005.166

Pitié F, Kokaram AC, Dahyot R (2007) Automated colour grading using colour distribution transfer. Computer Vision and Image Understanding 107(1-2):123–137, DOI 10.1016/j.cviu.2006.11.011

Scinocca JF, Kharin VV, Jiao Y, Qian MW, Lazare M, Solheim L, Flato GM, Biner S, Desgagne M, Dugas B (2016) Coordinated global and regional climate modeling. Journal of Climate 29:17–35, DOI 10.1175/JCLI-D-15-0161.1

Sun Y, Solomon S, Dai A, Portmann RW (2006) How often does it rain? Journal of Climate 19:916–934, DOI 10.1175/JCLI3672.1

Table S1: Event attribution results for many extreme fire risk metrics, including 90% confidence intervals. Values are rounded to two decimal places for display purposes.

Event	p_0		p_1		PN		PS		RR	
**Fire Season 90th percentile**										
FWI > 30	<0.01	-	0.03	[0.02, 0.04]	0.83	[0.67, 0.94]	0.03	[0.01, 0.04]	5.97	[3.07, 15.69]
FFMC > 91	0.05	[0.04, 0.07]	0.15	[0.13, 0.17]	0.66	[0.56, 0.75]	0.11	[0.08, 0.13]	2.95	[2.27, 3.97]
DMC > 60	0.23	[0.21, 0.26],	0.36	[0.32, 0.39]	0.35	[0.25, 0.44]	0.17	[0.11, 0.21]	1.55	[1.33, 1.77]
DC > 425	0.39	[0.36, 0.42]	0.56	[0.53, 0.59]	0.31	[0.24, 0.38]	0.29	[0.22, 0.34]	1.45	[1.32, 1.60]
ISI > 15	<0.01	-	<0.01	-	-	-	-	-	-	-
BUI > 90	0.15	[0.12, 0.17]	0.26	[0.23, 0.29]	0.44	[0.32, 0.54]	0.14	[0.09, 0.17]	1.78	[1.48, 2.17]
**Significant Spread Potential**										
> 38 days	0.03	[0.02, 0.04]	0.12	[0.10, 0.14]	0.74	[0.62, 0.84]	0.09	[0.07, 0.12]	3.90	[2.61, 6.10]
> 25%	0.04	[0.03, 0.05]	0.11	[0.09, 0.13]	0.65	[0.50, 0.77]	0.08	[0.05, 0.10]	2.82	[2.00, 4.18]
ROS p90 > 18 [C2]	<0.01	-	<0.01	-	1.00	-	<0.01	-	10^9	[10^7, 10^{12}]
**Fire Intensity Classes**										
> 38 days Class 5/6 [C2]	0.16	[0.14, 0.19]	0.37	[0.34, 0.40]	0.55	[0.47,0.62]	0.24	[0.20, 0.28]	2.22	[1.89, 2.60]]
> 76 days Class 5/6 [C2]	<0.01	-	<0.01	-	0.96	[0.78, 1.00]	<0.01	-	22.52	[4.61, 468.68]
HFI p90 > 10,000 [C2]	0.08	[0.06, 0.10]	0.22	[0.20, 0.25]	0.63	[0.53, 0.72]	0.15	[0.12, 0.18]	2.72	[2.14, 3.52]
**Fire Season**										
Fire season starts by 15 Apr	0.09	[0.07, 0.11]	0.19	[0.17, 0.22]	0.52	[0.39, 0.63]	0.11	[0.08, 0.14]	2.10	[1.64, 2.70]
Fire season ends after 31 Sep	0.09	[0.07, 0.10]	0.25	[0.22, 0.28]	0.65	[0.57, 0.72]	0.18	[0.14, 0.21]	2.86	[2.31, 3.64]
Fire season > 165 days	0.05	[0.04, 0.06]	0.20	[0.18, 0.23]	0.76	[0.67, 0.83]	0.16	[0.13, 0.19]	4.12	[3.04, 5.76]

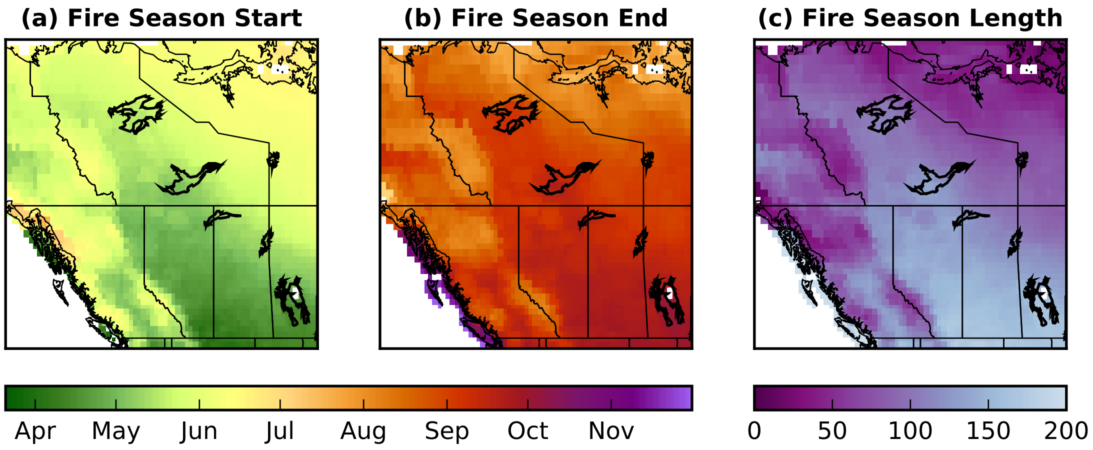

Figure S1: Average (1981-2010) fire season start date (a), end date (b), and fire season length in days (c) using snow data from GFWED. The start date occurs after three consecutive snow-free days (after 01 March) and the end date is the first day with snowfall recorded (after 01 July). Months are labelled on the first day.

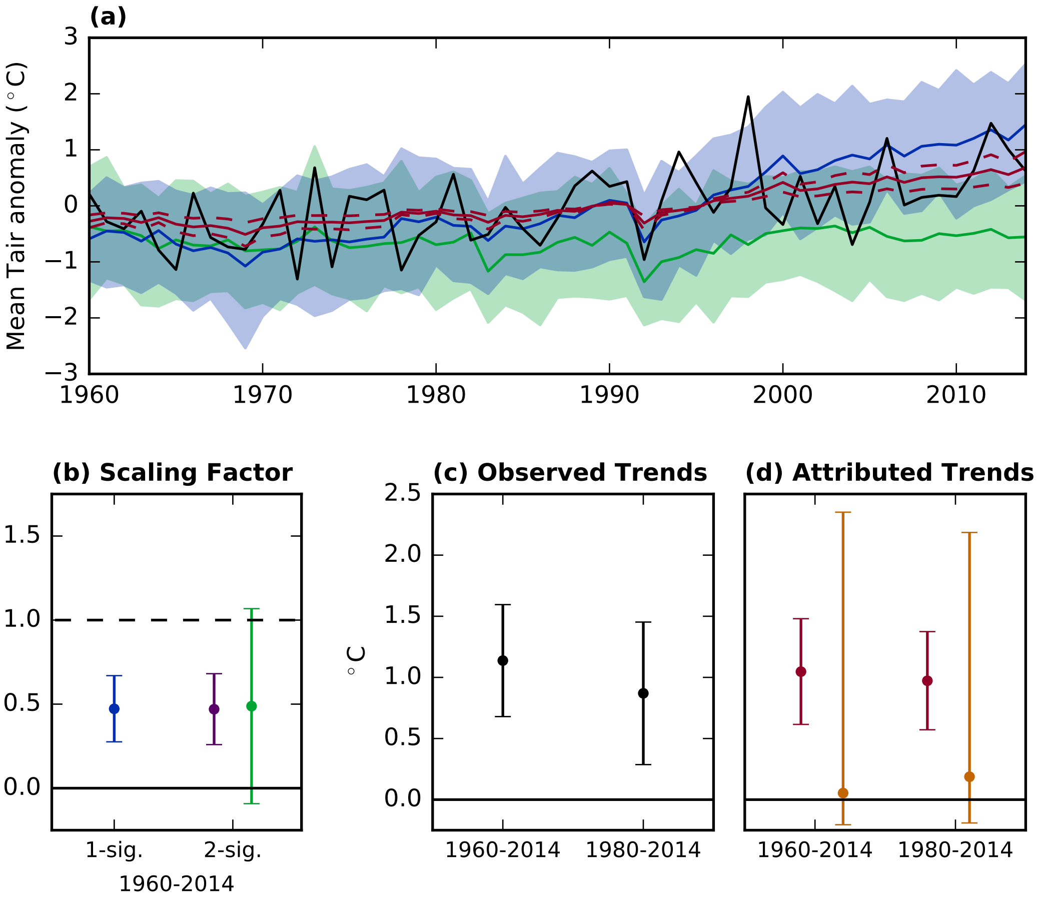

Figure S2: (a) Time series of anomalies in air temperature averaged over the May-Sep fire season for land grid boxes in western Canada for ALL forcing (blue), NAT forcing (green), and observations from CRU-TS3 (black). The ensemble means are plotted and shading indicates the 5th-95th percentile across the 50-member ensemble. In red is the ALL ensemble mean rescaled to match observations, with dashed 90% uncertainty. (b) Scaling factors and 90% confidence intervals from a one-signal detection and attribution analysis with ALL forcing (blue) and 2-signal for ANT (purple) and NAT (green). The one-signal scaling factor and uncertainty were used for the red lines in panel (a). (c) Observed trends in fire season mean temperature for two different time periods. Trends are assumed to be linear and uncertainty is determined from the regression analysis. (d) The resulting trend in fire season mean temperature that is attributed to the influence of ALL forcing (red), which is dominated by the influence of greenhouse gas increases from anthropogenic activities, and NAT forcing (orange). The rescaled ensemble means were used to calculate these trends.

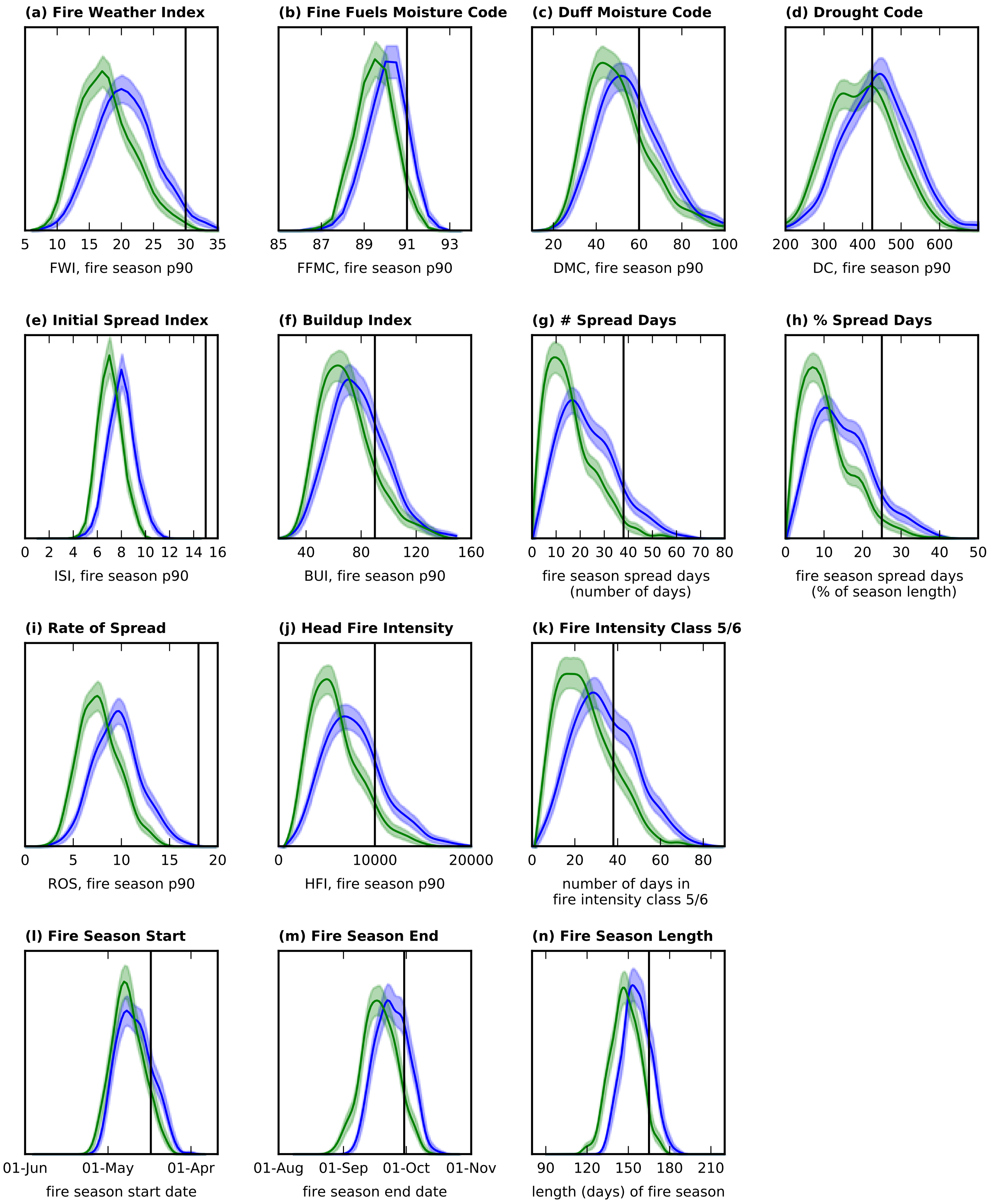

Figure S3: Density curves for many event metrics during 2011-2020 with ALL forcing in blue and NAT forcing in green. The uncertainty range for each curve is shaded and was calculated using a bootstrapping method. The FBP metrics in panels (i) - (k) use the C2 fuel class.

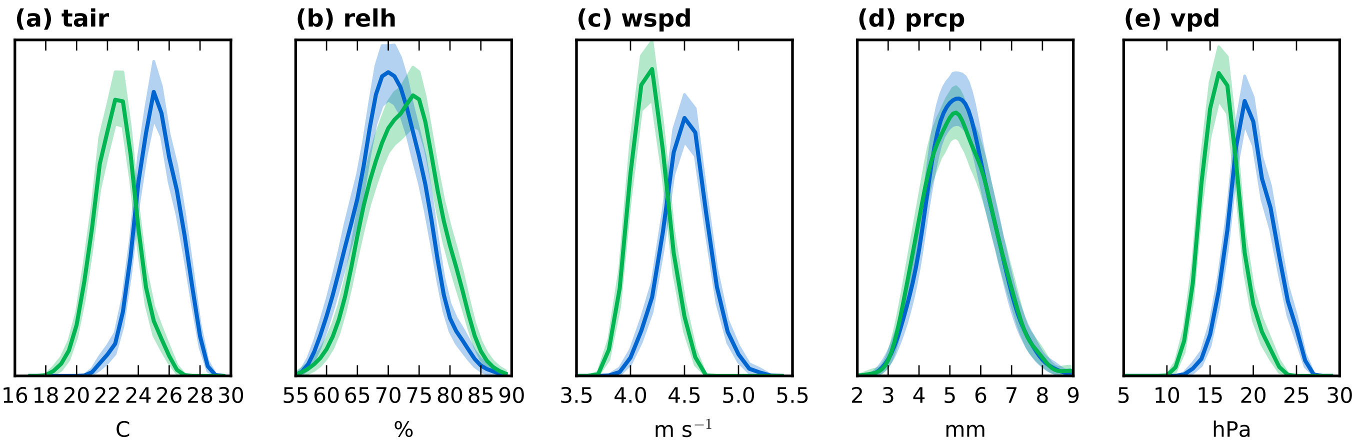

Figure S4: Density curves for the fire season 90th percentile value of the FWI System input variables and vapor pressure deficit (VPD) during 2011-2020 with ALL forcing in blue and NAT forcing in green. The uncertainty range for each curve is shaded and was calculated using a bootstrapping method.

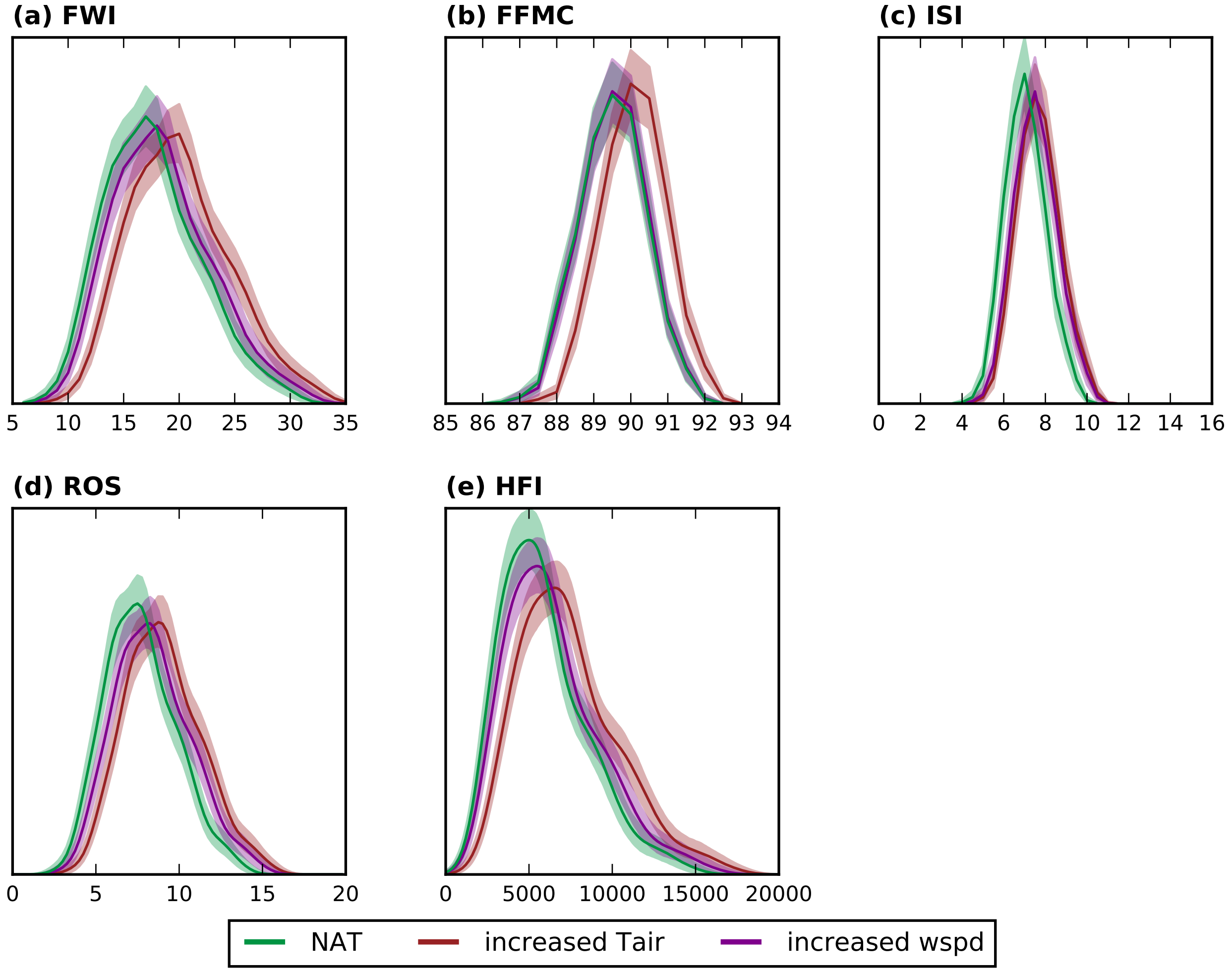

Figure S5: Density curves for the fire season 90th percentile value of the FWI and FBP indices during 2011-2020 averaged over HFR9 for NAT forcing shown in green. The red line is the same except the input temperatures were shifted to match the mean of the ALL forcing scenario. Purple line was calculated with wind speeds shifted similarly. The uncertainty range for each curve is shaded and was calculated using a bootstrapping method. Only indices that depend on wind speed are included. FBP metrics in panels (d) and (e) depend on the C2 fuel class.

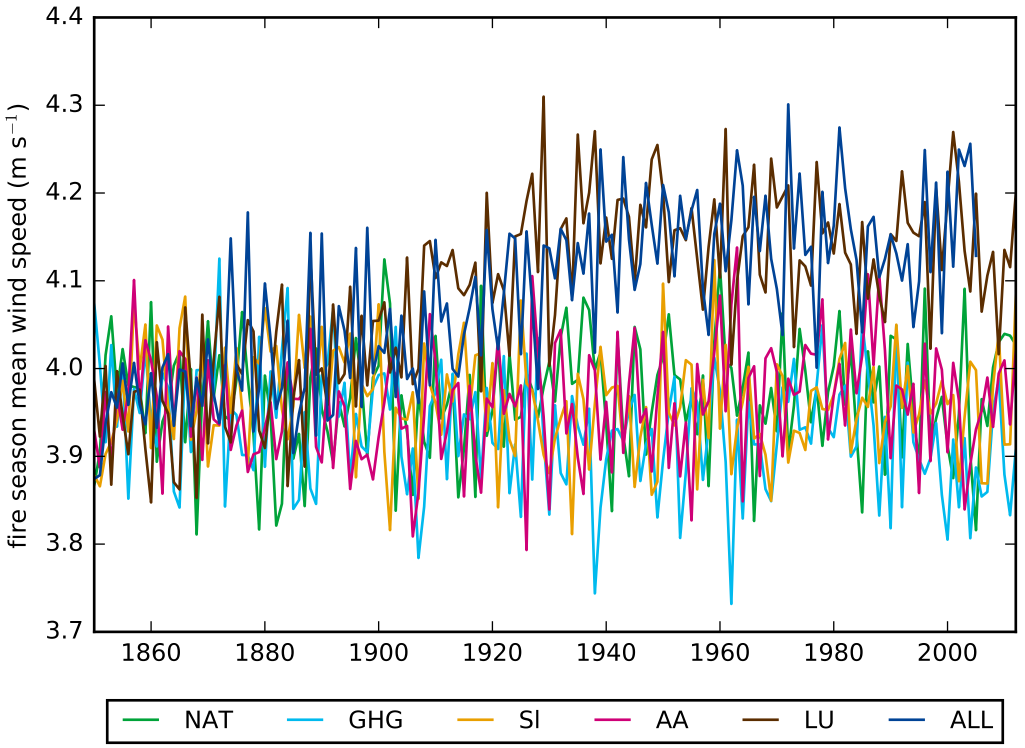

Figure S6: Mean wind speeds across HFR9 during the fire season (May - September) for CanEMS2 forced with natural-only (green), greenhouse-gas only (light blue), solar only (orange), atmospheric aerosols only (pink), land-use only (brown), and all forcings (dark blue). Values are averaged across the 5 ensemble members with data for the whole period plotted here.

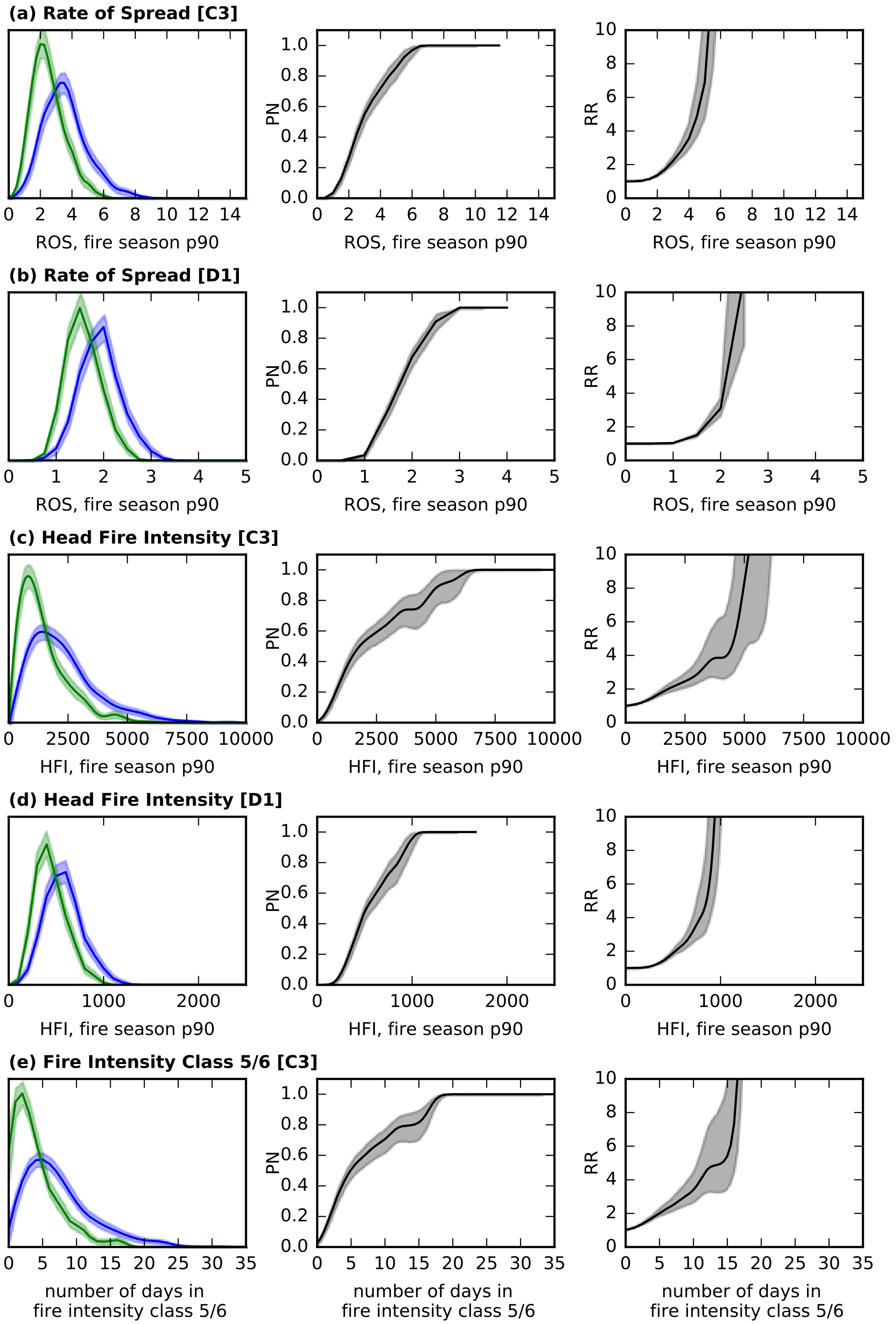

Figure S7: Density curves (left column), PN (center column), RR (right column) for the FBP metrics for fuel classes C3 and D1 during 2011-2020. D1 does not have any days in a fire intensity class of 5 or 6.

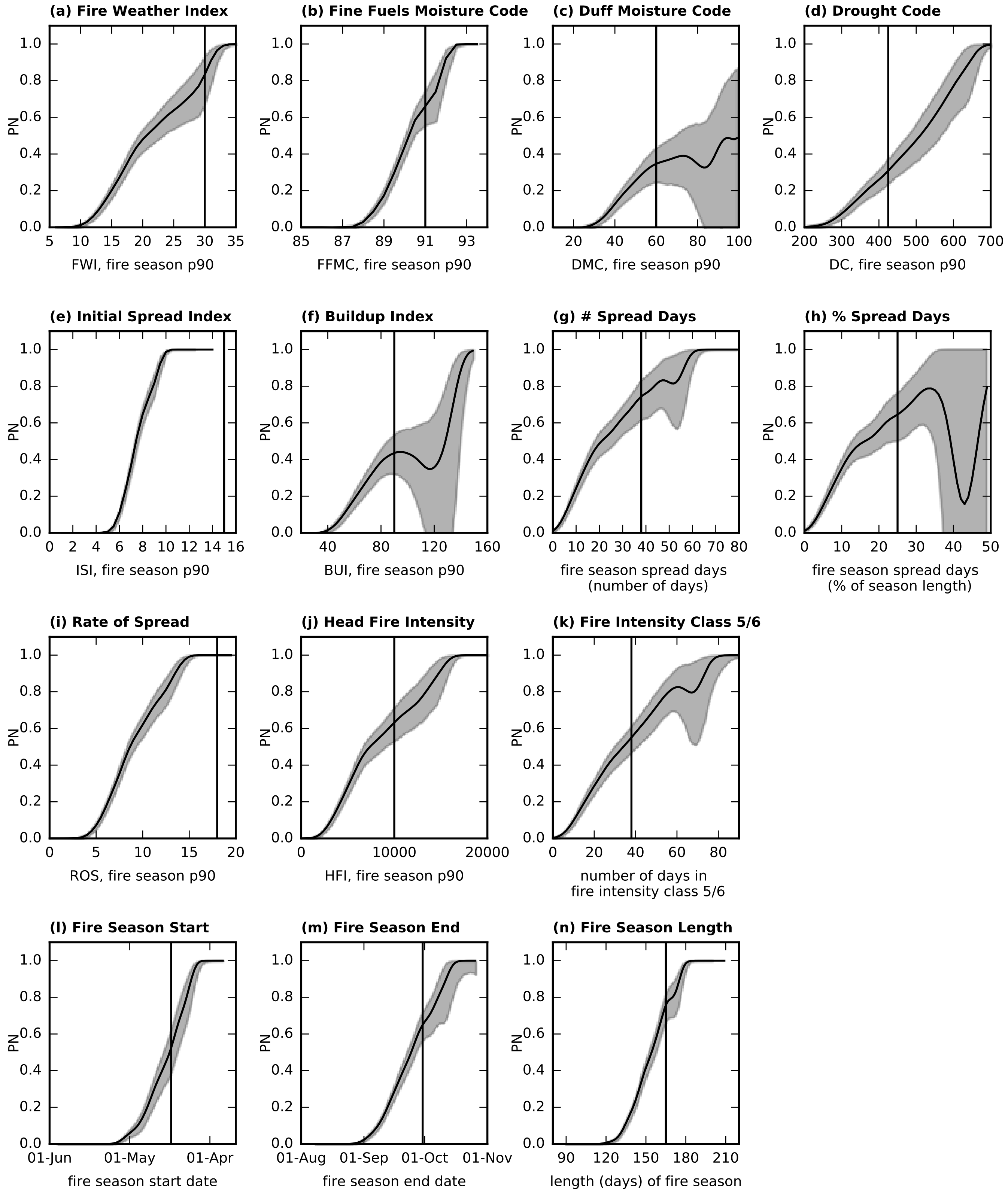

Figure S8: The probability of necessary causality (PN) for many event metrics using data for 2011-2020. Values are for an event more extreme than that indicated on the horizontal axis and the vertical bar represents the threshold for an extreme value. The uncertainty range for each PN curve is shaded and was calculated using a bootstrapping method. The FBP metrics in panels (i) - (k) use the C2 fuel class.
